# Supplementary material for: Comparing Bayesian spatial models: Goodness-of-smoothing criteria for assessing under- and over-smoothing
Source: PLoS One. 2020 May 20;15(5):e0233019. doi: 10.1371/journal.pone.0233019 (PMC7239453; doi:10.1371/journal.pone.0233019)

**Fig G:** Maps showing the posterior mean estimates of the key model parameters for the BYM model variants with an IG hyperprior (SIDS data set, 100 counties in North Carolina).


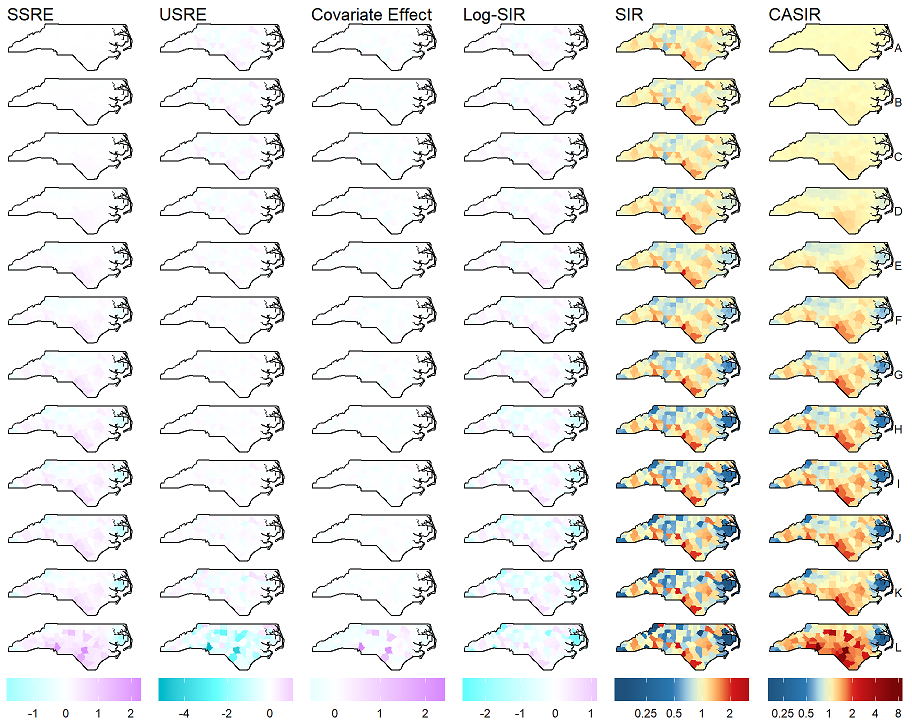

Supplement: S7 Fig — (DOCX) [file pone.0233019.s007.docx]
