# Supplementary figures and images for: Comparing Bayesian spatial models: Goodness-of-smoothing criteria for assessing under- and over-smoothing
Source: PLoS One. 2020 May 20;15(5):e0233019. doi: 10.1371/journal.pone.0233019 (PMC7239453; doi:10.1371/journal.pone.0233019)

**Fig I:** Model residuals for the lip cancer data set, 56 counties of Scotland.


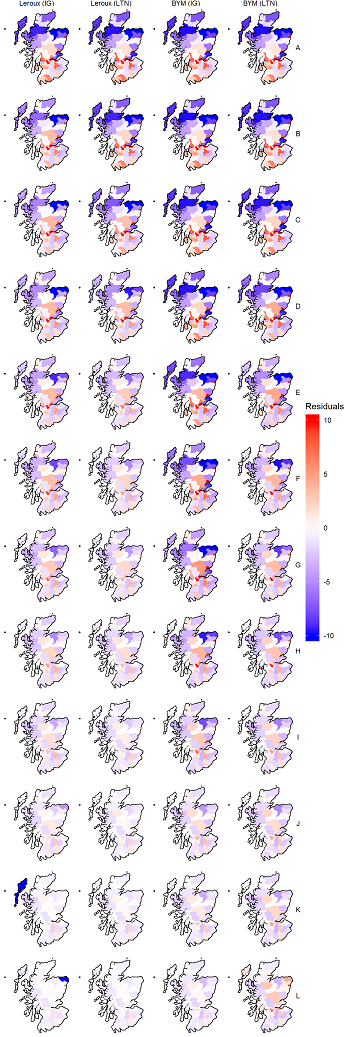

Supplement: S9 Fig — (DOCX) [file pone.0233019.s009.docx]

**Fig J:** Model residuals for the SIDS data set, 100 counties in North Carolina.


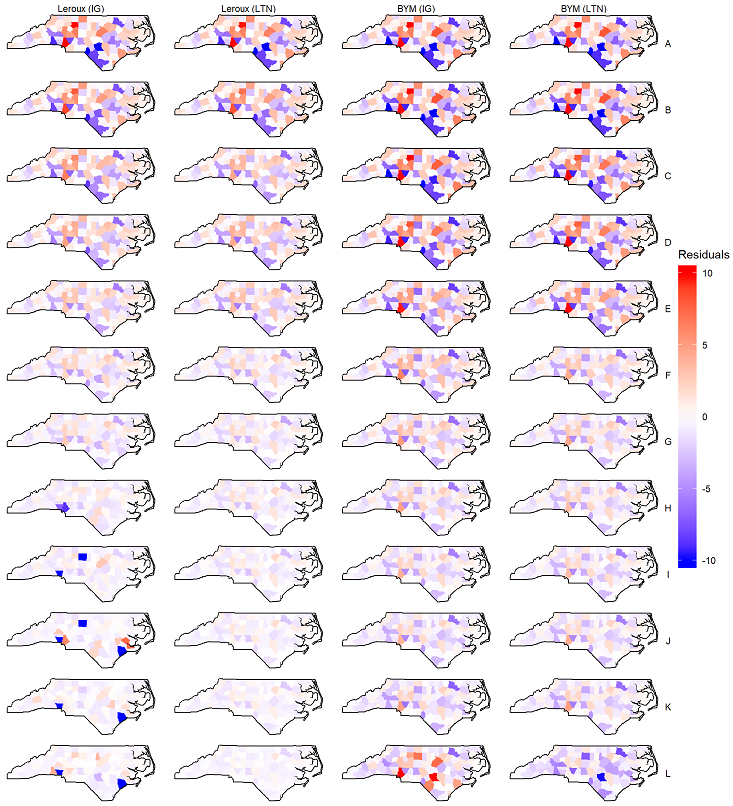

Supplement: S10 Fig — (DOCX) [file pone.0233019.s010.docx]

**Fig L:** Kurtosis and roughness for each model variant fit to the SIDS data set.


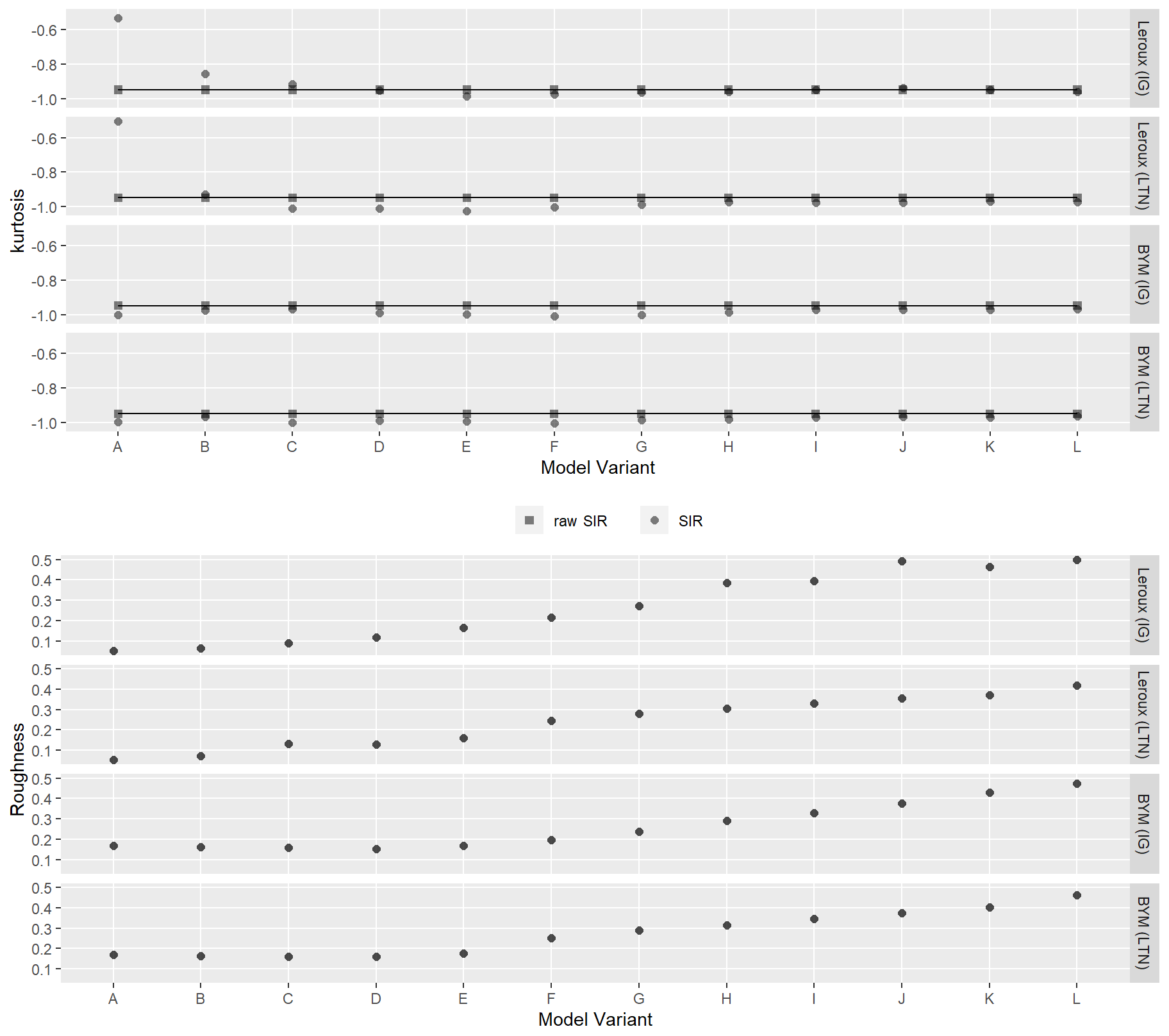

Supplement: S12 Fig — (DOCX) [file pone.0233019.s012.docx]

**Fig N:** Fraction of spatial variation for each model variant fit to the SIDS data set.


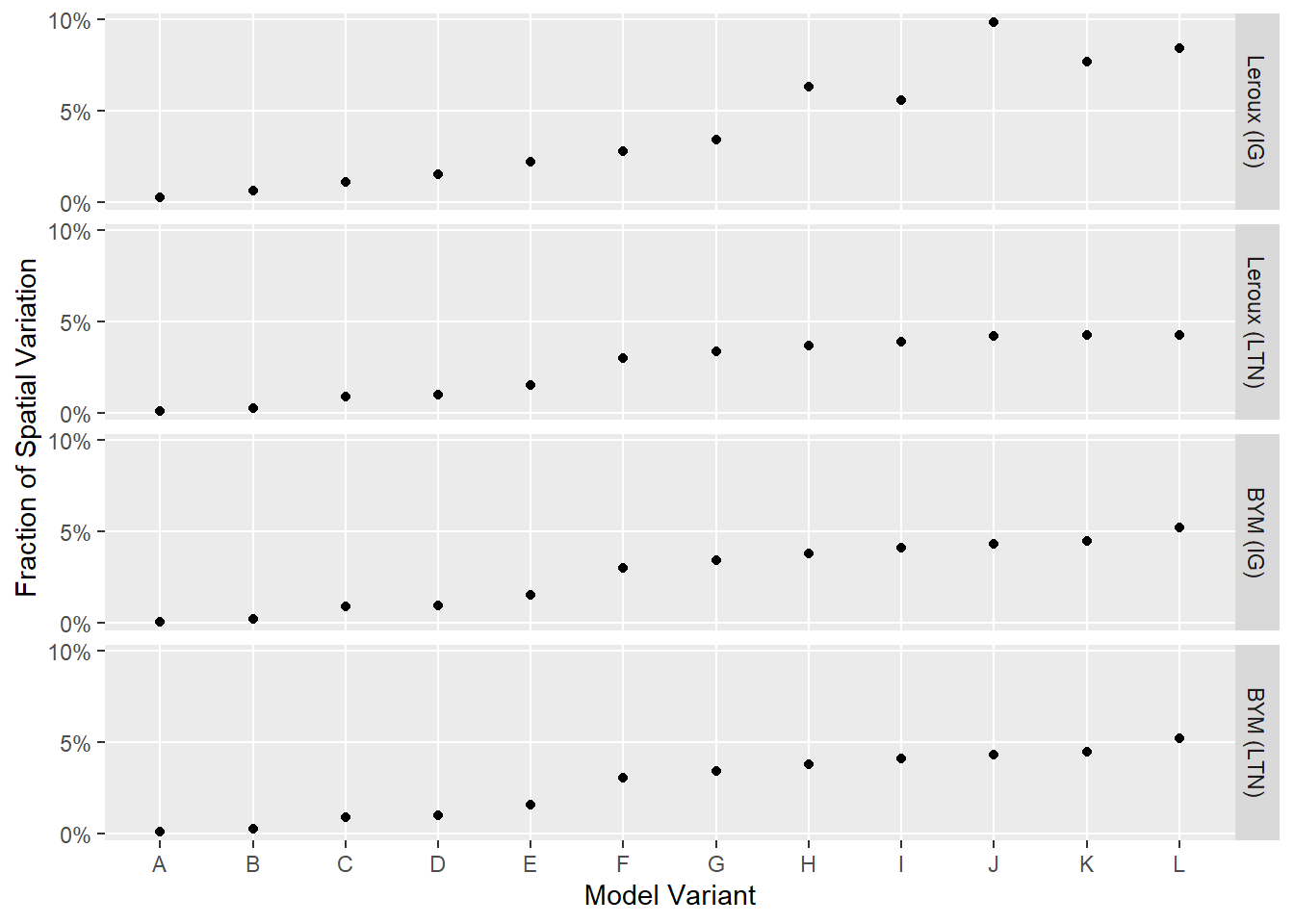

Supplement: S14 Fig — (DOCX) [file pone.0233019.s014.docx]
