# Supplementary material for: Comparing Bayesian spatial models: Goodness-of-smoothing criteria for assessing under- and over-smoothing
Source: PLoS One. 2020 May 20;15(5):e0233019. doi: 10.1371/journal.pone.0233019 (PMC7239453; doi:10.1371/journal.pone.0233019)

**Fig K:** Variograms for each model variant fit to the SIDS data set. The solid and dashed lines denote the variograms of CASIR and CARSIR respectively, each averaged over the areas. The grey dots denote the area-specific variogram of CASIR. Note that the y-axis has been capped at 1 for clarity.


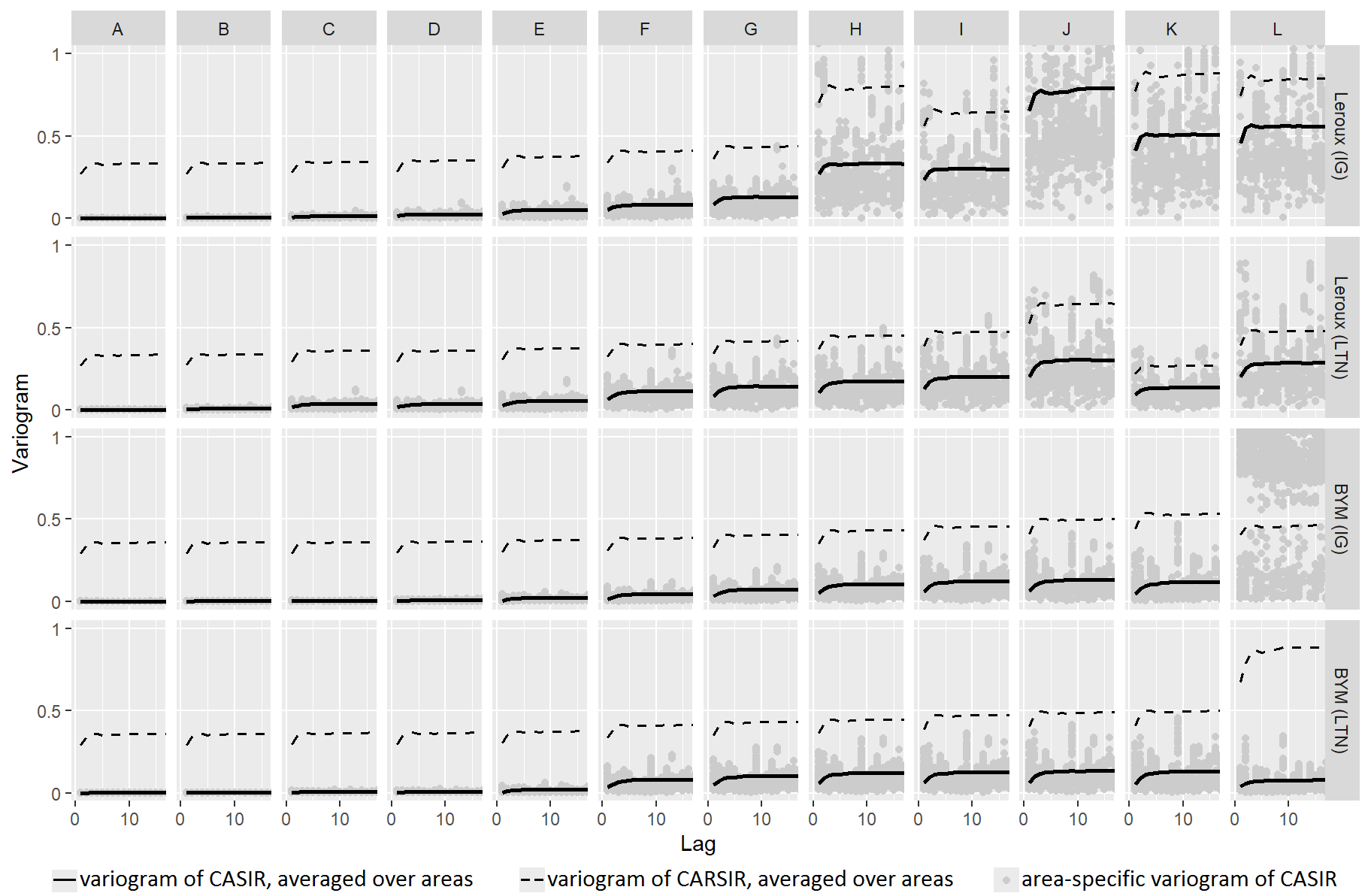

Supplement: S11 Fig — The solid and dashed lines denote the variograms of CASIR and CARSIR respectively, each averaged over the areas. The grey dots denote the area-specific variogram of CASIR. Note that the y-axis has been capped at 1 for clarity. (DOCX) [file pone.0233019.s011.docx]
