# Supplementary material for: Comparing Bayesian spatial models: Goodness-of-smoothing criteria for assessing under- and over-smoothing
Source: PLoS One. 2020 May 20;15(5):e0233019. doi: 10.1371/journal.pone.0233019 (PMC7239453; doi:10.1371/journal.pone.0233019)

**Fig M:** Kappa statistic between CASIR and CARSIR for each model variant fit to the SIDS data set, using 3 and 5 discrete categories. Values close to 0 suggest over-smoothing while values close to 1 suggest under-smoothing.


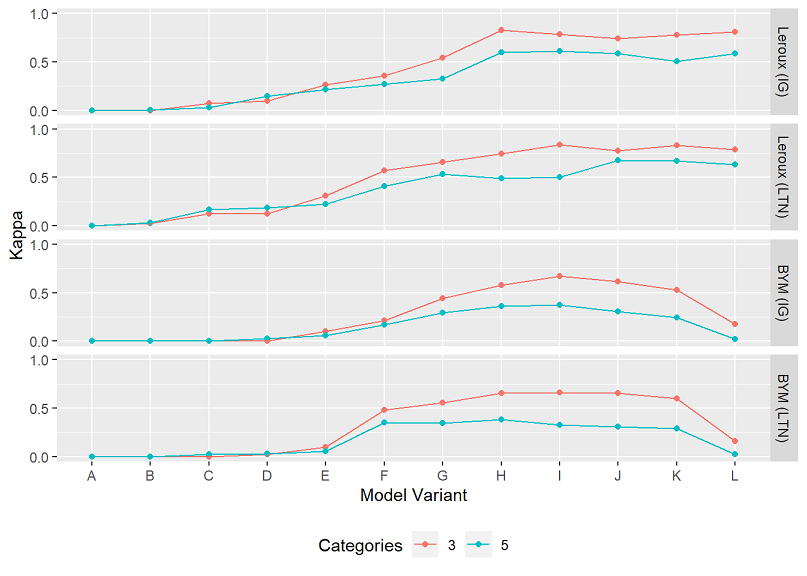

Supplement: S13 Fig — Values close to 0 suggest over-smoothing while values close to 1 suggest under-smoothing. (DOCX) [file pone.0233019.s013.docx]
