# Supplementary material for: Comparing Bayesian spatial models: Goodness-of-smoothing criteria for assessing under- and over-smoothing
Source: PLoS One. 2020 May 20;15(5):e0233019. doi: 10.1371/journal.pone.0233019 (PMC7239453; doi:10.1371/journal.pone.0233019)

**Fig O:** Distribution of the relative position of the CASIR estimates for each model variant (SIDS data). Distributions with substantial density close to 0 indicate under-smoothing; distributions with substantial density close to 1 indicate over-smoothing.


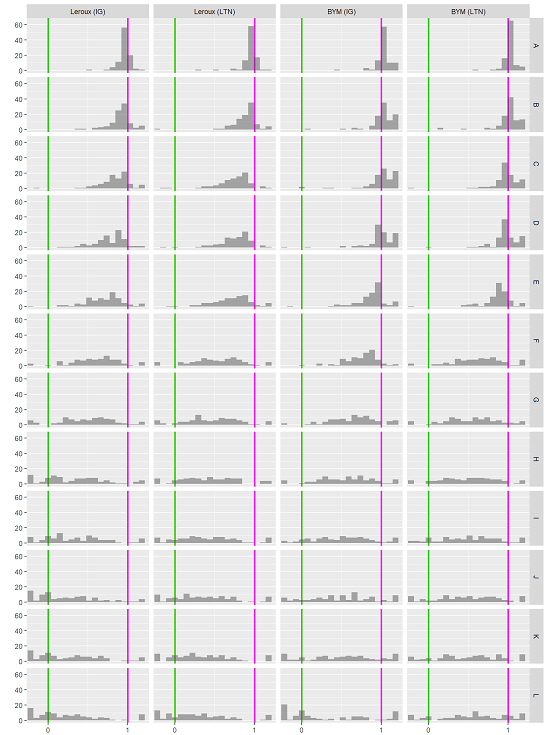

Supplement: S15 Fig — Distributions with substantial density close to 0 indicate under-smoothing; distributions with substantial density close to 1 indicate over-smoothing. (DOCX) [file pone.0233019.s015.docx]
