# Supplementary material for: Comparing Bayesian spatial models: Goodness-of-smoothing criteria for assessing under- and over-smoothing
Source: PLoS One. 2020 May 20;15(5):e0233019. doi: 10.1371/journal.pone.0233019 (PMC7239453; doi:10.1371/journal.pone.0233019)
Supplement: S1 Table — (DOCX) [file pone.0233019.s016.docx]

**Table A**: The specific values of the hyperparameters $\alpha$, $\eta$, $\nu$, and $\pi$ used to produce the model variants.

| Model variant | Lip cancer data | | | | SIDS | | | |
| --- | --- | --- | --- | --- | --- | --- | --- | --- |
|  | Inverse-gamma | | Left-truncated Normal | | Inverse-gamma | | Left-truncated Normal | |
|  | $\alpha$ | $\eta$ | $\pi$ | $\nu$ | $\alpha$ | $\eta$ | $\pi$ | $\nu$ |
| A | 20 | 0.05 | 0 | 10^-6^ | 20 | 0.2 | 0 | 0.0001 |
| B | 20 | 0.085 | 0 | 10^-5^ | 20 | 0.5 | 0 | 0.001 |
| C | 20 | 0.15 | 0 | 10^-4^ | 20 | 1 | 0 | 5 |
| D | 20 | 0.2 | 0 | 10^-3^ | 20 | 2 | 0.5 | 1 |
| E | 20 | 0.5 | 0 | 10^-2^ | 20 | 5 | 1 | 0.1 |
| F | 20 | 1 | 0 | 10^-1^ | 20 | 10 | 1.2 | 0.03 |
| G | 20 | 2 | 0.5 | 10^-1^ | 20 | 20 | 1.4 | 0.02 |
| H | 20 | 5 | 0.5 | 10^-2^ | 20 | 40 | 1.6 | 0.01 |
| I | 20 | 10 | 1 | 10^-3^ | 20 | 60 | 2 | 0.01 |
| J | 20 | 25 | 1.5 | 10^-4^ | 20 | 100 | 2.5 | 0.05 |
| K | 20 | 50 | 2 | 10^-5^ | 20 | 150 | 3 | 0.0001 |
| L | 20 | 125 | 3 | 10^-6^ | 20 | 200 | 5 | 0.0001 |
