# Supplementary material for: Comparing Bayesian spatial models: Goodness-of-smoothing criteria for assessing under- and over-smoothing
Source: PLoS One. 2020 May 20;15(5):e0233019. doi: 10.1371/journal.pone.0233019 (PMC7239453; doi:10.1371/journal.pone.0233019)
Supplement: S2 Table — A “PASS” indicates that the model variant is neither under- nor over-smoothing under the given criterion (see Table 1). VR = variogram ratio; KP = kurtosis preservation; K = kappa; RPC = relative position of CASIR; (u) = unbiased; (c) = conservative (less likely to choose under- or over-smoothed models); (pu) = penalise under-smoothing more heavily than over-smoothing. (DOCX) [file pone.0233019.s017.docx]

**Table B**: Classification of the models based on the GoS criteria (lip cancer data). A “PASS” indicates that the model variant is neither under- nor over-smoothing under the given criterion (see Table 1). Note: VR = variogram ratio; KP = kurtosis preservation; K = kappa; RPC = relative position of CASIR; (u) = unbiased; (c) = conservative (less likely to choose under- or over-smoothed models); (pu) = penalise under-smoothing more heavily than over-smoothing.

| Model | Test | A | B | C | D | E | F | G | H | I | J | K | L |
| --- | --- | --- | --- | --- | --- | --- | --- | --- | --- | --- | --- | --- | --- |
| Leroux (IG) | VR (u) |  |  |  |  | PASS | PASS | PASS | PASS | PASS | PASS | PASS | PASS |
|  | K (u) |  |  |  |  | PASS | PASS | PASS | PASS | PASS | PASS | PASS | PASS |
|  | RPC (u) |  | PASS | PASS | PASS | PASS | PASS | PASS | PASS | PASS | PASS | PASS |  |
|  | KP (u) |  | PASS | PASS | PASS | PASS | PASS | PASS | PASS | PASS | PASS |  |  |
|  | VR (c) |  |  |  |  |  | PASS | PASS | PASS | PASS | PASS | PASS |  |
|  | K (c) |  |  |  |  |  | PASS | PASS | PASS | PASS | PASS | PASS |  |
|  | RPC (c) |  |  | PASS | PASS | PASS | PASS | PASS | PASS | PASS | PASS |  |  |
|  | KP (c) |  |  |  |  |  | PASS | PASS | PASS | PASS |  |  |  |
|  | VR (pu) |  |  | PASS | PASS | PASS | PASS | PASS |  |  |  |  |  |
|  | K (pu) |  |  |  |  | PASS | PASS | PASS | PASS | PASS | PASS |  |  |
|  | RPC (pu) |  | PASS | PASS | PASS | PASS | PASS | PASS | PASS | PASS |  |  |  |
| Leroux (LTN) | VR (u) |  |  |  |  | PASS | PASS | PASS | PASS | PASS | PASS | PASS | PASS |
|  | K (u) |  |  |  |  | PASS | PASS | PASS | PASS | PASS | PASS | PASS | PASS |
|  | RPC (u) |  |  | PASS | PASS | PASS | PASS | PASS | PASS | PASS | PASS | PASS |  |
|  | KP (u) |  |  |  | PASS | PASS | PASS | PASS | PASS |  |  |  |  |
|  | VR (c) |  |  |  |  | PASS | PASS | PASS | PASS | PASS | PASS | PASS | PASS |
|  | K (c) |  |  |  |  | PASS | PASS | PASS | PASS | PASS | PASS |  | PASS |
|  | RPC (c) |  |  |  | PASS | PASS | PASS | PASS | PASS | PASS |  |  |  |
|  | KP (c) |  |  |  |  | PASS | PASS | PASS | PASS |  |  |  |  |
|  | VR (pu) |  |  |  | PASS | PASS |  |  |  |  |  |  |  |
|  | K (pu) |  |  |  |  | PASS | PASS | PASS | PASS | PASS |  |  |  |
|  | RPC (pu) |  |  |  | PASS | PASS | PASS | PASS | PASS |  |  |  |  |
| Model | Test | A | B | C | D | E | F | G | H | I | J | K | L |
| BYM (IG) | VR (u) |  |  |  |  |  |  |  | PASS | PASS | PASS | PASS | PASS |
|  | K (u) |  |  |  |  |  |  |  | PASS | PASS | PASS | PASS | PASS |
|  | RPC (u) |  |  |  |  |  |  |  |  | PASS | PASS | PASS |  |
|  | KP (u) |  |  |  |  |  | PASS | PASS | PASS | PASS |  |  |  |
|  | VR (c) |  |  |  |  |  |  |  | PASS | PASS | PASS | PASS | PASS |
|  | K (c) |  |  |  |  |  |  |  | PASS | PASS | PASS | PASS |  |
|  | RPC (c) |  |  |  |  |  |  |  |  | PASS | PASS |  |  |
|  | KP (c) |  |  |  |  |  | PASS | PASS | PASS | PASS |  |  |  |
|  | VR (pu) |  |  |  |  |  |  | PASS | PASS | PASS |  |  |  |
|  | K (pu) |  |  |  |  |  |  |  | PASS | PASS | PASS |  |  |
|  | RPC (pu) |  |  |  |  |  |  |  |  | PASS | PASS |  |  |
| BYM (LTN) | VR (u) |  |  |  |  |  | PASS | PASS | PASS | PASS | PASS | PASS | PASS |
|  | K (u) |  |  |  |  | PASS | PASS | PASS | PASS | PASS | PASS | PASS | PASS |
|  | RPC (u) |  |  |  |  |  |  | PASS | PASS | PASS | PASS | PASS |  |
|  | KP (u) |  |  |  | PASS | PASS | PASS |  |  |  |  | PASS |  |
|  | VR (c) |  |  |  |  |  | PASS | PASS | PASS | PASS | PASS | PASS | PASS |
|  | K (c) |  |  |  |  |  | PASS | PASS | PASS | PASS | PASS | PASS | PASS |
|  | RPC (c) |  |  |  |  |  |  |  | PASS | PASS |  |  |  |
|  | KP (c) |  |  |  | PASS | PASS | PASS |  |  |  |  |  |  |
|  | VR (pu) |  |  |  |  | PASS | PASS | PASS | PASS |  |  |  |  |
|  | K (pu) |  |  |  |  | PASS | PASS | PASS | PASS | PASS | PASS | PASS | PASS |
|  | RPC (pu) |  |  |  |  |  |  | PASS | PASS | PASS |  |  |  |
