# Supplementary material for: Comparing Bayesian spatial models: Goodness-of-smoothing criteria for assessing under- and over-smoothing
Source: PLoS One. 2020 May 20;15(5):e0233019. doi: 10.1371/journal.pone.0233019 (PMC7239453; doi:10.1371/journal.pone.0233019)
Supplement: Attachment — Submitted filename: Response to Reviewers.docx [file pone.0233019.s019.docx]

**Responses to Points Raised by the Editor**

**Comment 1**: Please ensure that your manuscript meets PLOS ONE's style requirements, including those for file naming. The PLOS ONE style templates can be found at http://www.plosone.org/attachments/PLOSOne_

formatting_sample_main_body.pdf and http://www.plosone.org/attachments/PLOSOne_formatting_sample_title_authors_affiliations.pdf

**Response 1**: The supporting information file has been renamed as “S1_appendix.pdf”. The marked-up and unmarked versions of the manuscript for resubmission have been named “Revised Manuscript with Track Changes” and “Manuscript” as required. The authors believe the rest of the manuscript meets PLOS ONE’s style requirements, including font, headings, spacing, and line numbering. Note that acronyms and abbreviations are defined upon first appearance in the text as required, but these are also listed at the top of the manuscript before the introduction. The authors are happy for the editors to omit this list if it does not fit PLOS ONE’s format.

**Comment 2**: We note that Figures 2-3 and S1-S10 in your submission contain map images which may be copyrighted. All PLOS content is published under the Creative Commons Attribution License (CC BY 4.0), which means that the manuscript, images, and Supporting Information files will be freely available online, and any third party is permitted to access, download, copy, distribute, and use these materials in any way, even commercially, with proper attribution. For these reasons, we cannot publish previously copyrighted maps or satellite images created using proprietary data, such as Google software (Google Maps, Street View, and Earth). For more information, see our copyright guidelines: <http://journals.plos.org/plosone/s/licenses-and-copyright>. We require you to either (1) present written permission from the copyright holder to publish these figures specifically under the CC BY 4.0 license, or (2) remove the figures from your submission:`

**Response 2**: All the maps presented in this manuscript and supporting material, namely Figures 2 and 3, and Figures S1-S10, have not been previously published. These maps were created by the authors using freely available shapefiles. Specifically, the shapefile used to produce the Scotland maps (Figures 2, 3, S1-S4, S9) is available from the R package CARBayesdata and the GeoDa website (<https://geodacenter.github.io/data-and-lab>), amongst other sources. Similarly, the shapefile used to produce the North Carolina maps (Figures 2, S5-S8, S10) is also available from the GeoDa website, and the R package spData, amongst others. Aside from using these shapefiles as inputs, the maps are solely the work of the authors. The authors are content to have our work published under the CC BY 4.0 license.

**Comment 3**: Please include captions for your Supporting Information files at the end of your manuscript, and update any in-text citations to match accordingly. Please see our Supporting Information guidelines for more information: http://journals.plos.org/plosone/s/supporting-information.

**Response 3**: The supporting information files have been renamed as Table A, Table B, Fig A, Fig B etc. as items in S1 Appendix as per the guidelines. The in-text citations have been updated accordingly. The caption for this single supporting file has been added to the manuscript as advised, namely “S1 Appendix: Supplementary Figures and Tables”.

**Reviewer 1**

**Comment 1**: As a researcher of traffic statistical analysis, I fully understand that Bayesian spatial models are widely used in accident statistical modelling (some recent references are listed below). It is suggested that latest development and applications for Bayesian spatial models (such as used in traffic fields) can be briefly introduced.

Apostolos Z, George Y. A review of spatial approaches in road safety. Accident Analysis and Prevention. https://doi.org/10.1016/j.aap.2019.105323.

Zeng Q, Gu W , Zhang X, et al. Analyzing freeway crash severity using a Bayesian spatial generalized ordered logit model with conditional autoregressive priors. Accident Analysis and Prevention, 2019, 127, 87-95.

Zeng Q, Wen H, Huang H, et al. A Bayesian spatial random parameters Tobit model for analyzing crash rates on roadway segments[J]. Accident Analysis and Prevention, 2017, 100: 37-43.

Ma Q, Yang H Xie K, et al. Taxicab crashes modeling with informative spatial autocorrelation. Accident Analysis and Prevention, 2019, 131, 297-307.

**Response 1**: The authors thank the reviewer for these useful references. We found the first and third reference particularly relevant for the motivation of this research and have added these to the introduction as suggested, on line 81.

**Comment 2**: The abstract is suggested to rewritten with four parts: objective, methods, results and conclusions.

**Response 2**: The abstract has been rewritten in four parts as suggested. The new abstract now reads:

Background:

Many methods of spatial smoothing have been developed, for both point data as well as areal data. In Bayesian spatial models, this is achieved by purposefully designed prior(s) or smoothing functions which smooth estimates towards a local or global mean. Smoothing is important for several reasons, not least of all because it increases predictive robustness and reduces uncertainty of the estimates. Despite the benefits of smoothing, this attribute is all but ignored when it comes to model selection. Traditional goodness-of-fit measures focus on model fit and model parsimony, but neglect “goodness-of-smoothing”, and are therefore not necessarily good indicators of model performance. Comparing spatial models while taking into account the degree of spatial smoothing is not straightforward because smoothing and model fit can be viewed as opposing goals. Over- and under-smoothing of spatial data are genuine concerns, but have received very little attention in the literature.

Methods:

This paper aims to demonstrates the problem with spatial model selection based solely on goodness-of-fit, to propose by proposing several methods for quantifying the degree of smoothing, and to compare these methods. Several commonly used spatial models are fit to real data, and subsequently compared using the against goodness-of-fit and goodness-of-smoothing statistics on real data.

Results:

The proposed goodness-of-smoothing statistics show substantial agreement in the task of model selection, and tend to avoid models that over- or under-smooth. Conversely, the traditional goodness-of-fit criteria often don’t agree, and can lead to poor model choice. In particular, the well-known deviance information criterion tended to select under-smoothed models.

Conclusions:

Some of the goodness-of-smoothing methods may be improved with modifications and better guidelines for their interpretation. However, these proposed goodness-of-smoothing methods offer researchers a solution to spatial model selection which is easy to implement. Moreover, they highlight the danger in relying on goodness-of-fit measures when comparing spatial models.

**Reviewer 2**

**Comment 1**: The commonly used inverse-Gamma (α,η) prior for variance parameter is sensitive to the values of α and η if the true variance is close to zero (Gelman, 2006). In addition to specifying different combination of values of α and η to fit models with varying degrees of smoothing, the authors are therefore suggested to use a uniform (0, M) prior for σ_s as a benchmark.

**Response 1**: The authors implemented model variants using a uniform (0, M) prior as suggested. Specifically, the BYM and Leroux models were re-run on both data sets, each using variants of the uniform prior by changing the value of M, ranging from 0.5 to 10^4 (larger values resulted in priors too vague for the sampler to produce samples from). The results showed very little variation between model variants. This seems to be consistent with Gelman (2006), namely that for a finite but sufficiently large M, inferences are not sensitive to this choice of M. Unfortunately, there is no guarantee that such models will produce a model with an adequate degree of smoothing. In the case of the Scottish lip cancer, the models with the uniform prior were actually quite good in terms of the goodness-of-smoothing compared to the best inverse-gamma and left-truncated normal priors. This was reflected by the GoS criteria as well as a visual inspection of the risk surface and associated maps. Conversely, for the North Carolina SIDS data, the models with the uniform prior resulted in slight over-smoothing for the Leroux variants (comparable to variant D of the Leroux IG and LTN models), and severe over-smoothing for the BYM variants (comparable to variants C of the BYM IG and LTN models). It is comforting to see the proposed GoS criteria performing as expected for the uniform model variants. However, due to the inability to produce variation in the results because of the insensitivity to the hyper –parameter, we have not included these results in the paper. The authors do agree with the reviewer that trying other model specifications would be useful, but this has already been noted as a limitation and a recommendation for future research (lines 805-806 of the revised manuscript).

**Comment 2**: The definition of spatial correlation should have an effect on the degree of smoothing. Despite simplification and easy to manipulate, use of binary first-order adjacency weights that only areas with common borders are assumed to be spatially correlated is indeed a strong assumption, especially in empirical case studies without validation. This limitation should be highlighted at the end of manuscript.

**Response 2**: The authors acknowledge that this is a strong assumption. The authors have stated this assumption up front in the introduction as one of the three constraints imposed on this study. Given the focus of the study is on proposing and comparing the goodness-of-smoothing criteria, we do not consider it a limitation of this study per se, but rather that consideration of other spatial weights specifications are out of scope. Consequently, no changes have been made to the manuscript.
